# Spike-Independent Infection of Human Coronavirus 229E in Bat Cells

**DOI:** 10.1128/spectrum.03483-22

**Published:** 2023-05-18

**Authors:** Marcus G. Mah, Martin Linster, Dolyce H. W. Low, Yan Zhuang, Jayanthi Jayakumar, Firdaus Samsudin, Foong Ying Wong, Peter J. Bond, Ian H. Mendenhall, Yvonne C. F. Su, Gavin J. D. Smith

**Affiliations:** a Programme in Emerging Infectious Diseases, Duke-NUS Medical School, Singapore; b Bioinformatics Institute, Agency for Science, Technology, and Research, Singapore; c Department of Biological Sciences, National University of Singapore, Singapore; d Centre for Outbreak Preparedness, Duke-NUS Medical School, Singapore; e SingHealth Duke-NUS Global Health Institute, SingHealth Duke-NUS Academic Medical Centre, Singapore; f Duke Global Health Institute, Duke University, Durham, North Carolina, USA; Erasmus MC

**Keywords:** evolution, pandemic, receptor usage, zoonotic, spike, 229E, coronavirus, receptor

## Abstract

Bats are the reservoir for numerous human pathogens, including coronaviruses. Despite many coronaviruses having descended from bat ancestors, little is known about virus-host interactions and broader evolutionary history involving bats. Studies have largely focused on the zoonotic potential of coronaviruses with few infection experiments conducted in bat cells. To determine genetic changes derived from replication in bat cells and possibly identify potential novel evolutionary pathways for zoonotic virus emergence, we serially passaged six human 229E isolates in a newly established *Rhinolophus lepidus* (horseshoe bat) kidney cell line. Here, we observed extensive deletions within the spike and open reading frame 4 (ORF4) genes of five 229E viruses after passaging in bat cells. As a result, spike protein expression and infectivity of human cells was lost in 5 of 6 viruses, but the capability to infect bat cells was maintained. Only viruses that expressed the spike protein could be neutralized by 229E spike-specific antibodies in human cells, whereas there was no neutralizing effect on viruses that did not express the spike protein inoculated on bat cells. However, one isolate acquired an early stop codon, abrogating spike expression but maintaining infection in bat cells. After passaging this isolate in human cells, spike expression was restored due to acquisition of nucleotide insertions among virus subpopulations. Spike-independent infection of human coronavirus 229E may provide an alternative mechanism for viral maintenance in bats that does not rely on the compatibility of viral surface proteins and known cellular entry receptors.

**IMPORTANCE** Many viruses, including coronaviruses, originated from bats. Yet, we know little about how these viruses switch between hosts and enter human populations. Coronaviruses have succeeded in establishing in humans at least five times, including endemic coronaviruses and the recent severe acute respiratory syndrome coronavirus 2 (SARS-CoV-2). In an approach to identify requirements for host switches, we established a bat cell line and adapted human coronavirus 229E viruses by serial passage. The resulting viruses lost their spike protein but maintained the ability to infect bat cells, but not human cells. Maintenance of 229E viruses in bat cells appears to be independent of a canonical spike receptor match, which in turn might facilitate cross-species transmission in bats.

## INTRODUCTION

Coronaviruses (CoVs) are positive-sense RNA viruses with large genomes ranging from 27 kb to 32 kb. CoVs are members of the *Coronaviridae* family, within the *Orthocoronavirinae* subfamily, and are divided into four genera, *Alphacoronavirus*, *Betacoronavirus*, *Deltacoronavirus*, and *Gammacoronavirus* (1). Four seasonal CoVs circulate globally, 229E, NL63, OC43, and HKU1 (2). Together, they account for 10% to 30% of upper respiratory tract infections in adults (3). Recently, three zoonotic coronaviruses have emerged in human populations, severe acute respiratory syndrome coronavirus (SARS-CoV) (4, 5), Middle East respiratory syndrome coronavirus (MERS-CoV) (6), and SARS-CoV-2 (7). Based on genetic analyses, 229E, NL63, SARS-CoV, SARS-CoV-2, and MERS-CoV have ancestral origins in bats (8, 9). Recently, 229E-related bat coronaviruses have been detected in *Hipposideros* and *Rhinolophus* bats across Africa (10, 11). Temporal analyses of 229E sequences from bats and humans have estimated that 229E viruses diverged from bat ancestral viruses more than 130 years ago (12). The detection of 229E-related viruses in captive alpaca and dromedary suggests that 229E might have evolved from bats to humans via camelids as intermediate hosts (13, 14).

Bats harbor a variety of pathogenic viruses, including Ebola virus, Nipah virus, Hendra virus, and coronaviruses, without displaying marked morbidity (15, 16). To infect humans, bat-derived CoVs need to overcome host-species barriers and balance genomic conservation and novel acquisition of genes with maintaining levels of infectivity, replication, and spread (17). Entry of CoVs into host cells is generally mediated by the binding of the spike protein to host receptors, which, in humans, comprise angiotensin-converting enzyme 2 (ACE2) for SARS-CoV (18), SARS-CoV-2 (19), and NL63 (20); dipeptidyl peptidase-4 (DPP4) for MERS-CoV (21); and aminopeptidase N (APN) for 229E (22). Also, sialic acid is the main attachment factor for OC43, HKU1 (23), and other animal alphacoronaviruses, such as porcine epidemic diarrhea virus (PEDV) and transmissible gastroenteritis virus (TGEV) (24). Genomic and functional studies have highlighted the flexibility of the spike protein to mutate and retain its capacity to bind to surface receptors. Due, in part, to the diversity of bats and coronaviruses, the exact receptor and entry mechanisms have not been identified. Here, we show evidence for the replication of human 229E CoVs that have lost spike gene expression during serial passaging in a kidney cell line derived from *Rhinolophus lepidus* bats (Blyth’s horseshoe bat). These viruses remain capable of infecting bat cells but are unable to infect human cells.

## RESULTS

**FIG 1:**
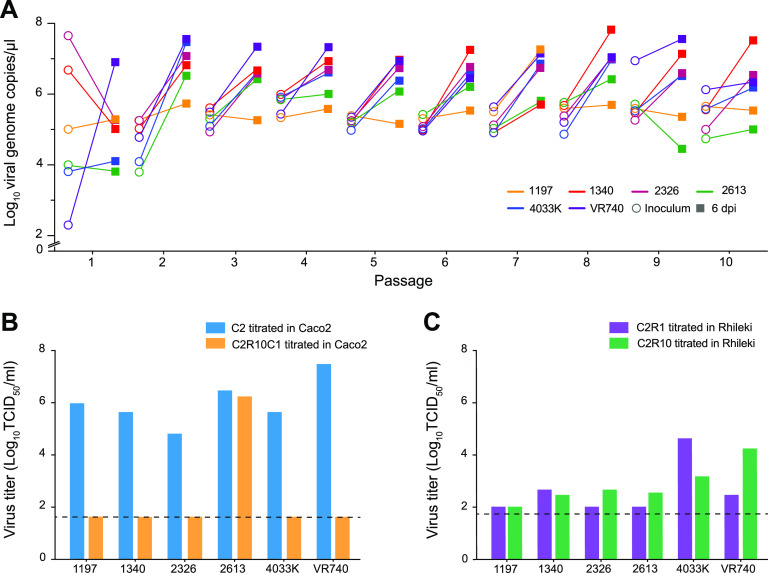
Rhileki cells are susceptible and permissive to 229E infection. (A) Serial passage of six 229E isolates at an MOI of 0.01 in Rhileki cells. Viral genome copies of the inoculum (circles) and at 6 days postinoculation (square) are plotted. The inoculum for subsequent passages was standardized based on genome copy numbers. (B) Viral titers (TCID_50_/mL) in Caco2 cells of C2, C2R10, and C2R10C1 viruses after serial passage. (C) Viral titers (TCID_50_/mL) in Rhileki cells of C2R1 and C2R10 viruses after serial passage. Black dashed lines represent the limit of detection.

To identify possible adaptive mutations, five clinical isolates (SG/1197/2010, SG/1340/2011, SG/2326/2011, SG/2613/2011, and TZ/4033K/2017) of 229E coronavirus obtained from nasopharyngeal swabs and one reference strain (VR740) were cultured in human colon adenocarcinoma (Caco2) cells twice (C2 virus). These viruses were then serially passaged 10 times (C2R1 to C2R10) in a newly established *Rhinolophus lepidus* kidney (Rhileki) cell line (Fig. S1 in the supplemental material). During virus passaging in Rhileki cells, an increase in viral genome copies from inoculum to 6 days postinoculation (dpi) was observed except for isolate SG/1197/2010 (Fig. 1A). The inoculation of Caco2 and Rhileki cells with C2 viruses resulted in infection as detected by nucleocapsid immunofluorescence staining (Fig. S2) and titration in the respective cell lines (Fig. 1B and C). However, C2R10 viruses were only able to infect Rhileki cells but not Caco2 cells, except for isolate SG/2613/2011. Viral titers of C2 viruses in Caco2 cells ranged from 6.8 × 10^4^ to 3.2 × 10^7^ 50% tissue culture infective dose/mL (TCID_50_/mL) (Fig. 1B), whereas C2R1 virus titers in Rhileki cells ranged from 1 × 10^2^ to 2.68 × 10^4^ TCID_50_/mL, and C2R10 viruses displayed virus titers from 1 × 10^2^ to 1.58 × 10^4^ TCID_50_/mL (Fig. 1C). Interestingly, when C2R10 viruses were inoculated in Caco2 cells (C2R10C1), only isolate SG/2613/2011 produced a virus titer of 1.8 × 10^6^ TCID_50_/mL (Fig. 1B). As an additional proof of viral genomic replication, double-stranded RNA was detected at 1 dpi in Caco2 and Rhileki cells (Fig. S3).

**FIG 2:**
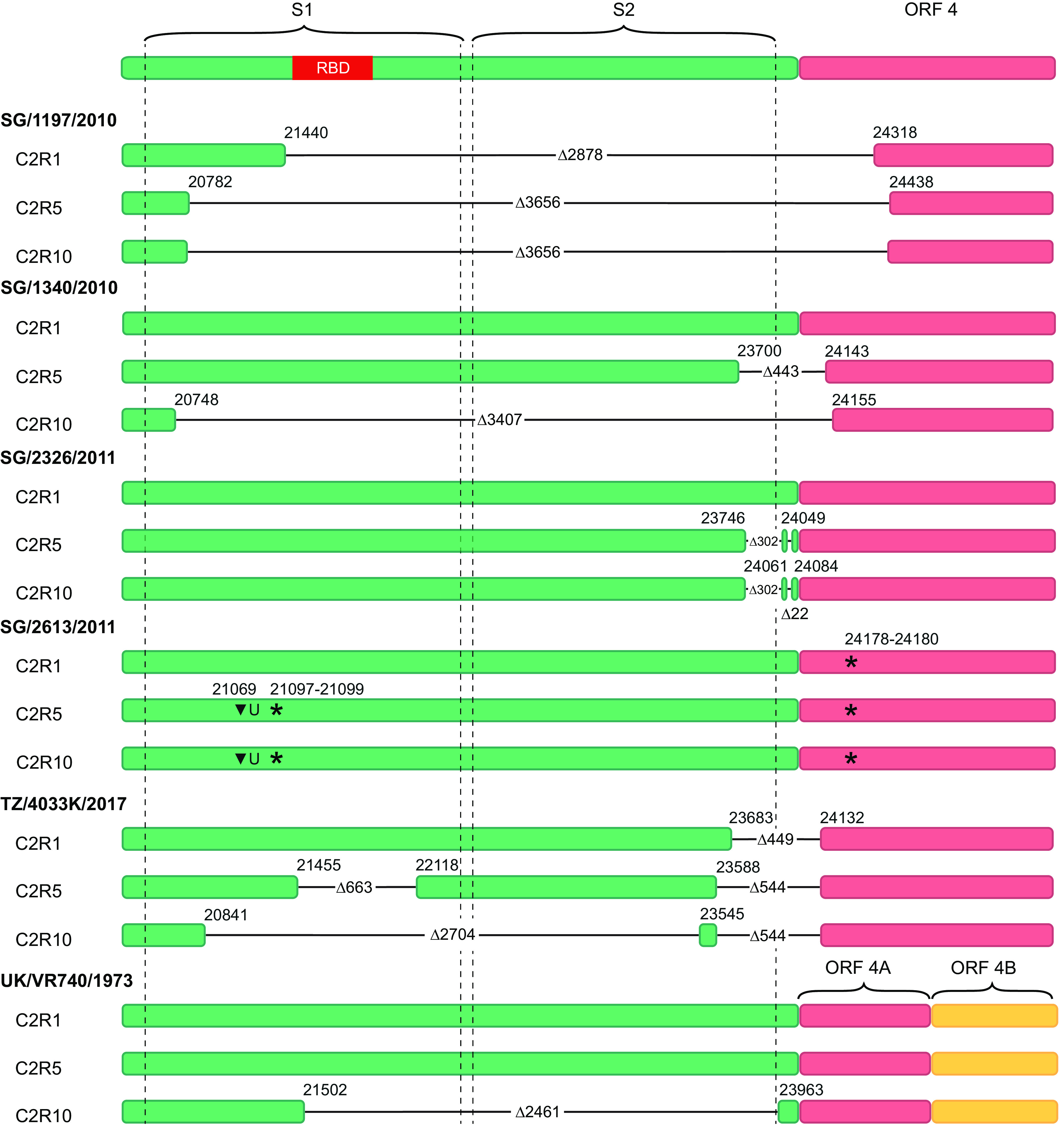
Schematic of nucleotide sequences within the spike and ORF4 gene regions. Color-filled rectangles represent parts of the genome in C2R1, C2R5, and C2R10 viruses that are present in the respective passage. Virus isolate names are in bold. Horizontal black lines indicate deleted parts of the genome with their respective size denoted by Δ; starts and ends of the deletions are indicated by the respective nucleotide position. Stop codons are denoted by an asterisk (*).

To determine the genetic changes that arose during passaging in Rhileki cells, we performed next-generation sequencing of C2R1, C2R5, and C2R10 viruses for the six 229E isolates (Fig. 2; Fig. S4). Surprisingly, deletions in the spike and ORF4 gene region were observed for SG/1197/2010 (2,878 nucleotides [nt]) and TZ/4033K/2017 (449 nt) during passage one. For SG/1340/2011 (443 nt) and SG/2326/2011 (302 nt and 22 nt), deletions were detected in the C2R5 virus. The reference strain UK/VR740/1973 displayed a deletion of 2,461 nt only during or before passage 10. No deletions were observed for SG/2613/2011. All deletions were confirmed by Sanger sequencing with isolate-specific flanking primers (Table S1), and minor variants were assessed by assembling reads that map to the deletions in the consensus sequences (Table S2). Potential viral subpopulations were detected in 0.65% to 2.64% of all reads. Between passage one and five, the deletion in SG/1197/2010 enlarged from 2,878 nt to 3,656 nt and remained constant after further passaging. Similarly, deletions in SG/1340/2011 extended from 443 nt to 3,407 nt between C2R5 and C2R10 viruses. While an additional deletion of 663 nt was found in isolate TZ/4033/2017 during or before passage five, which enlarged to 2,704 nt during or before passage 10, the initial deletion of 449 nt grew to 544 nt during or before passage five. Notably, the sequence encoding the receptor-binding domains (RBDs) in the spike gene were deleted in SG/1197/2010, SG/1340/2010, TZ/4033K/2017, and UK/VR740/1973. Although no deletions were observed for SG/2613/2011, there were nucleotide insertions at position 21069 that resulted in a premature stop codon at positions 21097 to 21099. Strikingly, as described above, the observed deletions and early stop codon did not prevent infection of Rhileki cells during the serial passaging, indicating that virus infection was occurring independent of the spike protein.

**FIG 3:**
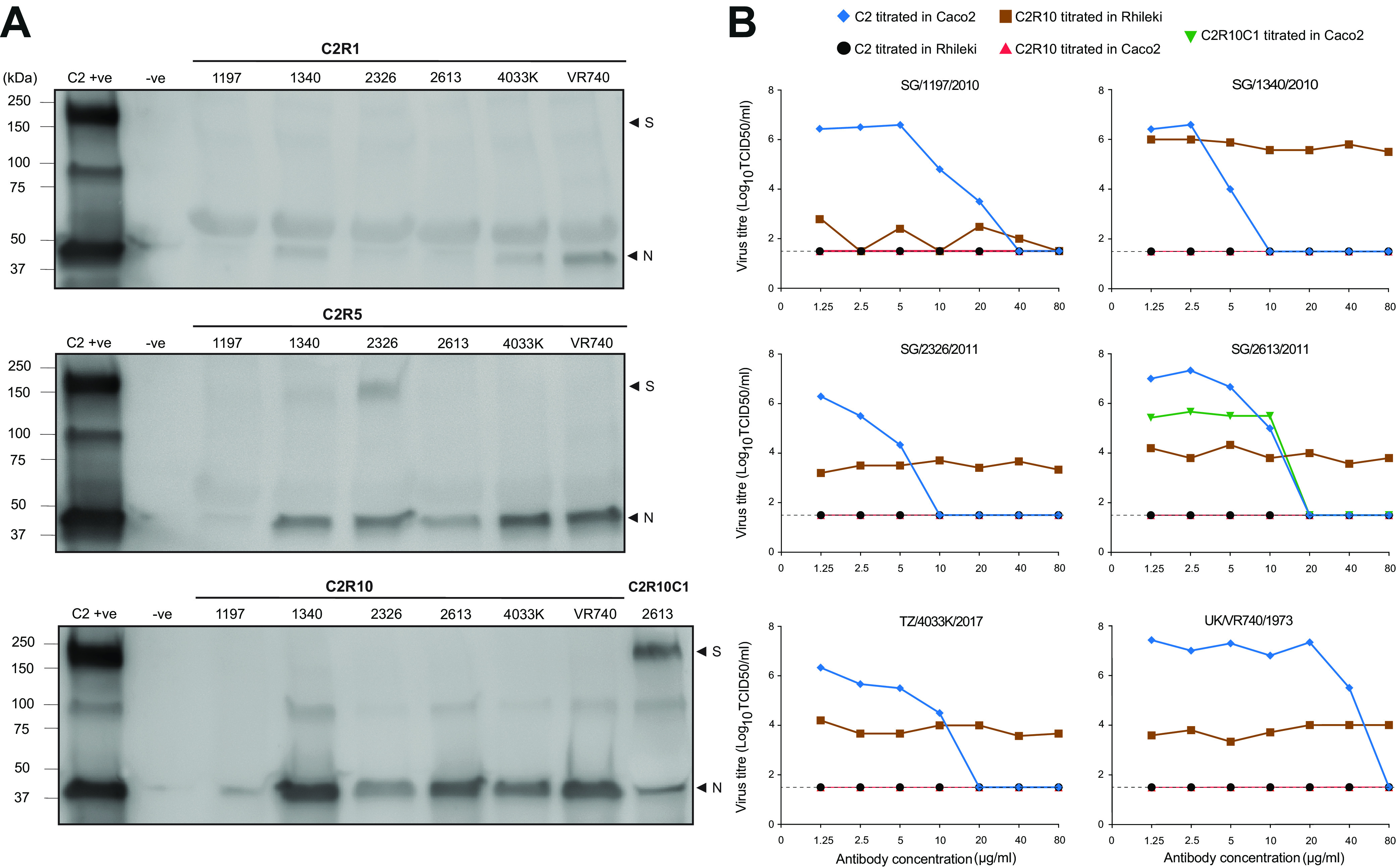
Spike protein expression and virus neutralization. (A) Western blot images of culture supernatants of Rhileki cells and Caco2 cells infected with C2R1, C2R5, C2R10, and C2R10C1 and stained with 229E anti-spike (S) and anti-nucleocapsid protein (N) antibodies. (B) Virus neutralization test, with virus titers (TCID_50_/mL) of C2 viruses in Caco2 cells (blue), C2 viruses in Rhileki cells (black), C2R10 viruses in Rhileki cells (brown), C2R10 viruses in Caco2 cells (red), and C2R10C1 virus in Caco2 cells for the isolate SG/2613/2011 only (green). The 229E anti-spike rabbit polyclonal sera at concentrations of 1.25 μg/mL to 80 μg/mL was incubated with the respective virus 1 h before inoculation. Blacked dashed lines indicate the limit of detection.

To verify the expression of spike protein in Rhileki cells, we generated a postimmunization polyclonal rabbit serum directed against the spike protein of 229E virus UK/VR740/1973 and determined the relative expression of spike protein of the serially passaged 229E isolates by Western blotting. No spike protein was detected in C2R1, C2R5, and C2R10 viruses, except for SG/2326/2011 C2R5 (Fig. 3A). Low levels of nucleocapsid protein were observed, particularly in C2R1 viruses, corresponding to the relatively lower virus titers observed in Rhileki cells. To test if continued viral growth in Rhileki cells was because of virus subpopulations (minor variants) that encode the spike protein, we performed virus neutralization tests using our 229E anti-spike polyclonal serum. Complete neutralization was achieved at 10 μg/mL for isolates SG/1340/2010 and SG/2326/2011, at 20 μg/mL for isolates SG/2613/2011 and TZ/4033K/2017, at 40 μg/mL for SG/1197/2010, and at 80 μg/mL for UK/VR740/1973 for C2 viruses in Caco2 cells (Fig. 3B). The six C2R10 viruses were able to infect Rhileki cells regardless of preincubation with spike-specific antibodies at average titers of 2.0 × 10^2^ TCID_50_/mL for SG/1197/2010, 3.2 × 10^3^ TCID_50_/mL for SG/2326/2011, 1.0 × 10^4^, 7.6 × 10^3^, and 6.8 × 10^3^ TCID_50_/mL for SG/2613/2011, TZ/4033K/2017, and UK/VR740/1973, respectively, and 6.4 × 10^5^ TCID_50_/mL for SG/1340/2010 at 3 days postinfection. The sole C2R10C1 virus SG/2613/2011 that was able to infect Caco2 cells was neutralized at 20 μg/mL, in line with its C2 counterpart. There were no detectable titers for C2 viruses in Rhileki cells or C2R10 viruses in Caco2 cells, although it should be noted that C2 viruses were passaged in Rhileki cells and are thus infecting at titers below the detection limit. Additionally, we used transmission electron microscopy to visualize purified C2 (Fig. S5A) and C2R10 (Fig. S5B) viruses. While spike protrusions were clearly present in C2 viruses, these were not observed in C2R10 viruses.

To assess if the spike and ORF4 deletions were also present on subgenomic mRNA (sgmRNA) transcripts, we probed the unique leader-body junction (LBJ) sequences of sgmRNA 2 and 4 (Table S1) extracted from total RNA of C2- and C2R10-infected Caco2 and Rhileki cells, respectively (Fig. S6A). While the expected PCR product size of 162 nt (sgmRNA 2) and 507 nt (sgmRNA 4) were obtained from C2 virus-infected Caco2 cells, there was variation in band sizes in C2R10 virus-infected Rhileki cells that correspond to the different-sized deletions detected in the viral genomic RNA (Fig. S6B). Sanger sequencing confirmed the presence of three types of LBJs. Canonical sgmRNA 2 LBJ was detected in all isolates, sgmRNA 4 LBJ was present in SG/2613/2011 and UK/VR740/1973. Interestingly, a non-canonical LBJ was detected only in SG/2326/2011 at nucleotides 23502 to 23558 of the spike open reading frame before the putative start codon for protein X.

Given the absence of spike protein detection in our previous assays, we visualized the remaining amino acids on the three-dimensional (3D) structures of the spike and ORF4 proteins using analogues from SARS-CoV-2 to probe whether the remaining sequences in C2R10 viruses could form a functional open reading frame (Fig. S7A). Only isolate SG/2326/2011 retained an intact spike ectodomain. However, the lack of transmembrane (TM) and heptad repeat 2 (HR2) regions that are involved in membrane fusion may impair spike function. The TM domain of ORF4 protein, a viroporin (25), is present in all viruses except isolate SG/1197/2010 (Fig. S7B).

To investigate the potential effect of mutations elsewhere in the viral genome, we identified single nucleotide polymorphisms (SNPs) in various passages (Table S3). Most SNPs (10/16) occurred in the nonstructural proteins 2, 3, 4, 8, 10, and 15. There were 15 SNPs that resulted in a nonsynonymous mutation, of which, 4 were fixed as the major variant at passage 1, 9 at passage 5, and all 15 at passage 10. Interestingly, isolates SG/1340/2010 and SG/2326/2011 acquired mutation A4854G (D621G in NSP3) independently, whereas SG/2326/2011 and TZ/4033K/2017 obtained C25177T (L54F in the membrane protein). In addition, SG/2613/2011 acquired mutation C24200T that resulted in an early stop codon (Q30* in ORF4). Further investigation is required to determine if these mutations have any biological effect.

**FIG 4:**
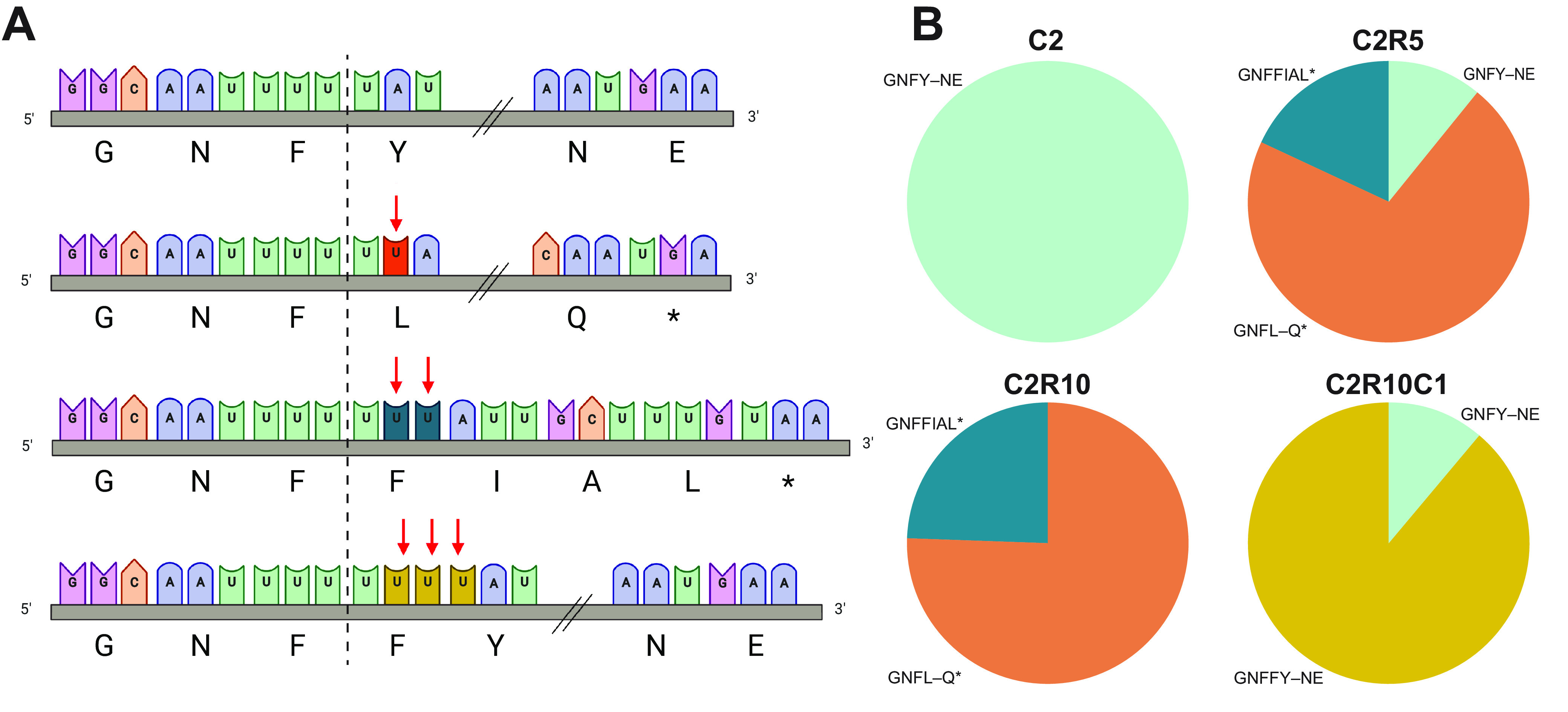
Genetic diversity of the SG/2613/2011 spike gene across passages. (A) Nucleotide sequences from position 21059 to 21100 and the corresponding amino acid translation within the S1 domain of the spike gene. Depending on the passage of the virus, the diversity is comprised of one to three uracil insertions at nucleotide position 21064 that alter the reading frame compared to the wild-type sequence (turquoise, line 1), GNFY-NE. With one (orange, line 2) or two (teal, line 3) uracil insertions, the reading frame shifts and leads to an early stop codon GNFL-Q* (E177*) and GNFFIAL* (L171*), respectively. At three (mustard, line 4) uracil insertions, the reading frame is restored with an additional phenylalanine GNFFY-NE. Double forward slashes (//) indicate 8 amino acid residues not shown for clarity of presentation. (B) Pie charts describe the percentage of viral genome reads with uracil insertions for C2 (100% no insertions, turquoise, *n* = 8,268), C2R5 (11.1% no insertions; 70.9% single insertion, orange; 18.0% double insertion, teal, *n* = 323), C2R10 (75.8% single insertion; 24.2% double insertion, *n* = 33), and C2R10C1 (11.2% no insertions; 88.8% triple insertion, mustard, *n* = 347) viruses. Figure was created with https://BioRender.com.

Only the C2R10 virus of isolate SG/2613/2011 resulted in infection of Caco2 cells (Fig. 1B), and the resulting C2R10C1 virus was positive for spike expression by Western blotting (Fig. 3A). Therefore, we examined the sequencing reads for the SG/2613/2011 spike gene, which did not display any deletions during virus passaging. Instead, virus subpopulations with insertions of 1 to 3 nt at position 21069 that resulted in loss and reinstatement of the spike open reading frame were observed (Fig. 4). The C2R5 virus population had three variants, the wild type (GNFY-NE), the majority variant (GNFL-Q*) with a 1-nt insertion resulting in a stop codon at positions 21097 to 21099, and a final variant (GNFFIAL*) with a 2-nt insertion and a stop codon at position 21078 to 21080. The wild type was not detected in C2R10, possibly due to low sequencing coverage (Fig. S4). For SG/2613/2011, the C2R10C1 virus that was grown in Caco2 cells, both the wild type (GNFY-NE) and a new variant (GNFFY-NE) with a 3-nt insertion that reinstates the spike open reading frame were present, reiterating that spike is essential for efficient virus infection of human cells.

**FIG 5:**
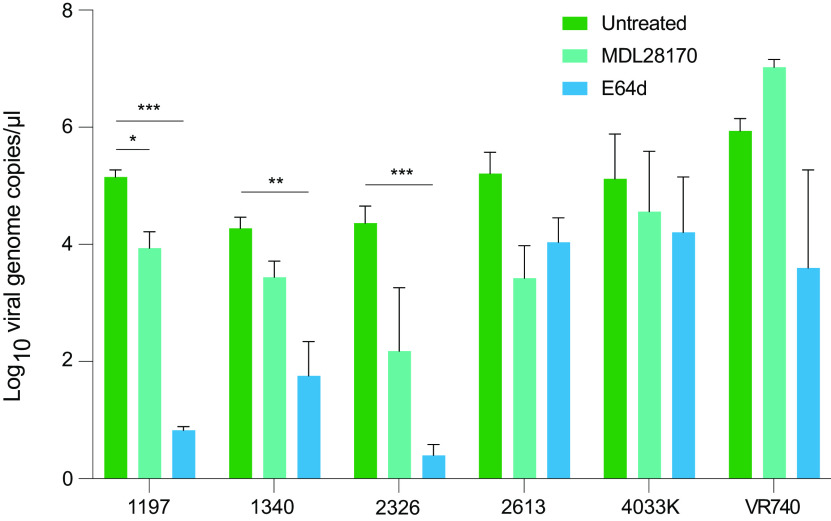
Effect of cathepsin inhibitors on C2R10 virus growth on Rhileki cells. Rhileki cells were pretreated with either 50 μM MDL28170 (turqoise) or 10 μM E64d (blue) before infection with C2R10 viruses at an MOI of 0.003. Viral genome copies were measured at 3 dpi. Cell viability towards a range of concentrations of the tested compounds were assessed. Cell viability: dimethyl sulfoxide (DMSO), 100%; 50 μM MDL28170, 89.3%; 10 μM E64d, 100%. The experiment was repeated three independent times and in triplicates. Error bars represent standard error of the mean; *, *P* = 0.0163; **, *P* < 0.005; ***, *P* < 0.0001.

As viruses derived from repeated virus passaging were able to infect Rhileki cells despite the absence of spike protein, cysteine proteases inhibitors MDL28170 and E64d were used to block cathepsin-mediated coronavirus endosomal entry (26, 27). Reduced replication was observed for all C2R10 viruses at 3 dpi in Rhileki cells treated with either MDL28170 or E64d compared to in untreated cells, except for MDL28170-treated cells inoculated with UK/VR740/1973 (Fig. 5). These findings suggest that C2R10 viruses likely use an undefined receptor to enter Rhileki cells via the endosomal pathway.

## DISCUSSION

Two independent mechanisms for cell entry by coronaviruses are known, receptor-dependent cathepsin-mediated endocytosis and transmembrane serine protease-mediated direct cell membrane entry (28). Both mechanisms rely on binding of viral spike protein to various cellular host receptors. Human sarbecoviruses, which include SARS-CoV and SARS-CoV-2, bind to ACE2 via residues within the RBD that are deleted or mutated in the majority of bat sarbecoviruses (29–32). Clade 1 sarbecoviruses (e.g., SARS-CoV, SARS-CoV-2, and bat CoVs like WIV1) can use human ACE2 for entry (30, 33). Clade 2 viruses (e.g., RmYN02, Rs4081, and Rf4092) contain deletions in the RBD that prevent binding to either human or bat ACE2. Clade 3 and clade 4 sarbecoviruses contain one deletion in the RBD and show a partial capacity to bind ACE2 orthologues in humans and bats (34). It has been recently shown that besides spike-receptor binding, protease activity is required to cleave the spike protein for entry (35). Despite the addition of exogeneous trypsin and expression of known CoV receptors, clade 2 sarbecovirus Rs4081 was unable to replicate in baby hamster kidney cells but infected intestine cells derived from of Rhinolophus sinicus, thus hinting at a yet unknown receptor in bats (34). Likewise, protein-protein binding assays have shown that the spike proteins of various bat sarbecoviruses were unable to bind bat ACE2 receptors (36, 37). Furthermore, receptor-independent entry of coronaviruses has been documented for murine hepatitis virus (MHV) (38), MERS-CoV (35), and SARS-CoV-2 (39). While spike-receptor compatibility and protease-mediated spike activation are barriers for cross-species transmission (35), growing evidence suggests alternative modes of coronavirus infection exist in bats.

To identify mutations arising from adaptation of human coronaviruses in bat cells, we serial passaged and sequenced human 229E viruses in Rhileki cells. Deletions in the spike and ORF4 regions occurred as early as the first passage. Critically, the expression of the spike protein was absent in all C2R10 viruses despite sustained replication in Rhileki cells. This intriguing finding was confirmed by Western blotting, virus neutralization, and electron microscopy, suggesting a spike-independent mode of entry into Rhileki cells. Virus titers of C2R10 viruses were consistently lower in Rhileki cells than C2 viruses in Caco2 cells, suggesting that high replicative fitness of CoVs in bats might not necessarily be an evolutionary advantage (40) and may not be required for virus maintenance in bat populations. Spike-independent modes of infection might help explain general features of CoV infection observed in bats, such as low levels of viremia (41) and a dampened immune response even to highly pathogenic viruses (42).

Bat sarbecoviruses that have deletions within the spike protein RBD and are unable to bind human or bat ACE2 were described (30, 34), adding to speculation that spike-independent infection could be mediated by other viral proteins, like envelope or membrane. Sialic acids are known attachment factors for other alphacoronaviruses (24); however, the sialic acid binding residues are not present in the S1 domain of the 229E spike protein (43). A potential viral protein resulting from an alternative 229E reading frame could mediate spike-independent entry into bat cells. Based on the usage of cysteine protease inhibitors E64D and MDL28170, we showed that 229E viruses passaged in Rhileki cells are using the endosomal route for entry, suggesting involvement of a receptor. Compared to the highly specific lock-and-key interaction of canonical spike-receptor engagement, spike-independent fusion of viral and cellular membranes would likely be less efficient. Whether this nonspecific mechanism of infection applies to all bat coronaviruses or is evolutionarily replaced by the more favorable spike-receptor engagement in other hosts requires further study.

This study reports the abrogation of spike protein expression consistently among six human 229E isolates in a bat kidney cell line. Although this phenomenon has not been observed in viruses isolated from bats, the extensive deletions in 229E might be due to the additional selective pressure for a relatively smaller genome size that is more pronounced in serial passaging cell culture experiments than in the repeated infection of an organism in nature. As an alternative to large genomic deletions, the insertion of one or two uracil nucleotides, leading to an early stop codon in isolate SG/2613/2011, was observed. This further suggests that spike protein is not required for virus maintenance in the bat cell line used. Interestingly, the insertion of a third uracil restored the reading frame to result in a spike protein with one additional amino acid after passaging in human cells, reiterating the importance of spike expression for infection of human cells. It is currently unclear if spike-independent infection is restricted to Rhileki cells, *Rhinolophus lepidus*, horseshoe bats, or other bat species or if it includes other groups of animals. Furthermore, it is not known if spike-independent infection is restricted to 229E, alphacoronaviruses, and bat-borne CoVs or if it presents an even more generic way of asymptomatic infection by a larger group of viruses. Future experiments are needed to establish spike-independent infection of 229E and other coronaviruses using bat *in vitro* and *in vivo* models to establish the infectivity of infectious virus clones with and without the spike protein.

## MATERIALS AND METHODS

### Establishment of the Rhileki cell line.

Kidney tissue of a *Rhinolophus lepidus* bat (NUS-IACUC B01/12) was processed into cellular mixtures by mechanical disruption through a 100-μm cell strainer. One hundred thousand cells were plated in a T-25 Corning CellBIND flask containing maintenance medium (RPMI with l-glutamine, 10% fetal bovine serum [FBS], 2.5% HEPES, and 1× antibiotics-antimycotics) at 37°C and 5% CO_2_ for expansion. Cells were then serially diluted in 384-well plates. Wells containing single-cell patches were selected, trypsinized, and serially diluted an additional two times before expansion. A spontaneously immortalized clonal cell line was selected and named Rhileki for *Rhinolophus lepidus* kidney, which is currently maintained until passage 30.

### Virus culture and serial passage.

Isolates SG/1197/2010, SG/1340/2010, SG/2326/2011, and SG/2613/2011 were obtained from a study in Singapore (44). Isolate TZ/4033K/2017 was obtained from a study conducted in Tanzania (unpublished). UK/VR740/1973 was procured from ATCC. All viruses were cultured for 3 days in Caco2 cells (ATCC, HTB-37) at 33°C in Dulbecco’s modified Eagle medium (DMEM), GlutaMAX, containing 3% FBS and 1% penicillin-streptomycin. Viruses were titrated for 5 days in a 96-well format, and plates were fixed with 100% methanol, blocked with 3% bovine serum albumin (BSA), and stained with 1E7 primary monoclonal antibody (Eurofins Ingenasa, Spain) binding to the 229E nucleocapsid protein and a goat anti-mouse secondary antibody conjugated with fluorescein isothiocyanate (Abcam, UK). TCID_50_ titers were calculated according to the method of Reed and Muench (45).

Rhileki cells were seeded at a density of 2 × 10^6^ in T25 flasks. The virus inoculum at a multiplicity of infection (MOI) of 0.01 was incubated at 33°C for 2 h. The inoculum was removed, cells were washed once with phosphate-buffered saline (PBS), and medium was replenished (DMEM, 1% sodium pyruvate, 1% nonessential amino acids, and 0.5% BSA). The supernatant was collected at time points 0, 3, 6, and 9 dpi. At 9 dpi, the cells were scraped, pelleted, and stored at −80°C. For serial passages, viral genome copies in the supernatant at 6 dpi were adjusted to contain 10^5^ to 10^6^ genome equivalents in the subsequent inoculum. Caco2 and Rhileki cells were seeded into two-well chambered coverglass on the day before infection. Caco2 and Rhileki cells were infected with C2 or C2R10 viruses, respectively, for 2 h at 33°C. At 1 dpi, cells were fixed and permeabilized with 4% paraformaldehyde and 0.1% Triton X-100, respectively. J2 antibody (Abcam, UK) was used to visualize double-stranded RNA.

### Endosome entry assay.

Rhileki cells were seeded in 24-well plates at a density of 3 × 10^5^ cells per well overnight. On the day of infection, cells were washed once with PBS and treated with medium, 50 μM MDL28170 (Sigma-Aldrich, USA), or 10 μM E64d (Tocris Bioscience, UK) for 1 h at 37°C. After pretreatment, cells were spin inoculated in medium containing either of the two compounds and C2R10 viruses at an MOI of 0.003 by centrifugation at 1,500 × *g* for 1 h at 4°C. Thereafter, the inoculum was removed, and cells were incubated with medium containing the corresponding chemical for 5 h before replenishment of medium without chemicals and incubation at 33°C for 72 h.

### Cell proliferation and viability assay.

Rhileki cells were seeded in 96-well plates at a density of 2 ×10^6^ cells per plate. The toxicity of compounds MDL28170 and E64d was assessed with a cell proliferation XTT kit (Merck, USA). Absorbance readings were obtained using a plate reader (Tecan, USA).

### RNA extraction and PCR.

Briefly, total RNA was extracted from 150 μL of supernatant using the Direct-zol RNA MiniPrep kit (Zymo, USA) according to manufacturer’s instructions. Samples were analyzed by real-time quantitative reverse-transcription PCR using primers specific for 229E nucleocapsid. Nucleocapsid sequences were cloned, and cycle threshold values were converted to viral genome copies per microliter based on regression analysis of plasmid dilutions.

### Whole-genome sequencing.

cDNA libraries of passages 1, 5, and 10 were constructed using a TruSeq RNA library prep kit (Illumina, USA) according to the manufacturer’s instruction. The quality of the libraries were verified using RNA Bioanlalyzer (Agilent, USA) and quantity normalized using the KAPA library prep kit (Roche, Switzerland). The libraries were sequenced using a 250-bp flow cell on an Illumina MiSeq System. Raw next-generation sequencing reads were trimmed by Trimmomatic v0.39 to remove adaptors and low-quality reads (46). The sequence of each C2 virus isolate was used as a reference to assemble trimmed and raw reads derived from Rhileki passages. The assembly was done using the BWA-MEM algorithm in UGENE v34 (47). Coverage, depth, and statistics of the assembled reads were visualized in Geneious R9.1.8 and Integrative Genomics Viewer (48). Consensus sequences were aligned using MAFFT v7.222 and annotated in Geneious R9.1.8.

### Genetic analysis.

Trimmed and untrimmed reads were compared to flag deleted regions in the genome. To confirm the deletions, flanking primers were designed (Table S1 in the supplemental material). Using isolate-specific primers, cDNA synthesis was performed using a Superscript III first-strand synthesis system (Thermo Fisher, USA). This was followed by PCR, where a 50-μL mixture consisted of cDNA, sense/antisense primers (10 μM), 10× reaction buffer, *Pfu* polymerase (Promega, USA), and 10 mM dNTP mix (Thermo Fisher, USA). The PCR was conducted at 95°C for 2 min, 35 cycles at 95°C for 1 min, 47°C for 30 s, and 72°C for 2 min, and a final extension at 72°C for 5 min. PCR products were visualized using a 1.5% agarose gel, and sequences were confirmed by Sanger sequencing (Macrogen, South Korea).

### Subgenomic mRNA Sanger sequencing.

As part of the replication cycle of CoVs, translation of structural proteins is preceded by discontinuous transcription of subgenomic RNA (sgRNA) (49), whereby nested species of subgenomic mRNA (sgmRNA) are produced. Structural proteins of 229E are translated from sgmRNA 2, 4, 5, 6, and 7, while sgmRNA 3 is truncated and was described to be nonfunctional (50–52). The leader-body junction consists of nucleotides within the transcription-regulating sequence of the leader (TRS-L) and of the body (TRS-B). The hybridization process is mediated by long-range RNA-RNA interactions that lead to the formation of sgmRNAs, which can be distinguished based on their sequence from the genomic RNA. Reverse primers specific for the spike (226R) and ORF4 (617R) genes were designed to synthesize cDNA from total RNA extracted from infected cell lysates. Using a seminested PCR approach, the 2F primer targeting the start of the leader RNA was used in combination with primer 158R and subsequently with primer 87R to generate PCR products that contain the leader RNA, leader-body junction, and a portion of sgmRNA 2. Similarly, to test the presence of sgmRNA 4, primer 2F was used together with primers 520R and 397R. PCR products were visualized on a 1.5% agarose gel.

### Protein modeling.

The full genomic sequences of human coronavirus 229E C2R10 isolates were translated to amino acid sequences using the Expasy Translate webserver (https://web.expasy.org/translate/) (53) in all three forward 5′ to 3′ reading frames. These amino acid sequences were then aligned to full-length sequences of 229E spike and ORF4 proteins obtained from GenBank (accession number NC_002645) using the Clustal Omega webserver (https://www.ebi.ac.uk/Tools/msa/clustalo/) (54) to determine reading frames corresponding to each protein and deleted residues. The full-length structural models of human coronavirus 229E spike protein were built using the SARS-CoV-2 spike protein model as the template (55). Modeller version 9.21 (56) was used to generate 10 homology models, and the best model was selected based on having the lowest discrete optimized protein energy (57), while minimizing Ramachandran outliers (58). Using similar protocols, the ORF4 protein model was built using the cryo-electron microscopy structure of the SARS-CoV-2 ORF3a ion channel (Protein Data Bank [PDB]: 7KJR) (59) as the template. The N- and C-terminal regions were unresolved and were therefore modeled as unstructured loops. Deleted regions of the C2R10 isolates were mapped to these structures and visualized in the Visual Molecular Dynamics software (60).

### Polyclonal serum generation and virus neutralization test (VNT).

Recombinant human coronavirus 229E spike protein (Sinobiological, China) was used to immunize two female 16-week-old New Zealand rabbits (Pizhou Dongfang, China) three times with 2-week intervals. Whole blood was subjected to antibody affinity purification. Caco2 and Rhileki cells were seeded at a concentration of 2 × 10^6^ cells before infection. Polyclonal antisera were serially diluted 2-fold and were incubated with 126 TCID_50_ of C2, C2R10, and C2R10C1 viruses for 1 h at 33°C. Subsequently, Caco2 and Rhileki cells were inoculated for 2 h at 33°C, medium was replenished, and virus titers were quantified at 3 dpi.

### Western blotting.

Supernatants harvested from infected Rhileki cells were centrifuged at 3,500 rpm and precipitated with 8% polyethylene glycol (PEG) 8000 (Merck, Germany) overnight in 4°C. The solution was centrifuged at 12,000 rpm for 1 h at 4°C, and pellets were resuspended in NTE buffer (1 M NaCl, 1 M Tris-HCl [pH 8.0], and 0.5 M EDTA [pH 8.0]). The suspension was subjected to a 20% sucrose cushion via ultracentrifugation using a SW41 rotor at 32,000 rpm for 2 h at 4°C. The pellet was resuspended in NTE buffer and reduced with 5× lane marker sample buffer (Thermo Fisher, USA) at 95°C for 10 min. Purified virions were separated on a 4 to 20% Mini-PROTEAN TGX stain-free protein gel (Bio-Rad, USA). The gel was transferred to a polyvinylidene fluoride membrane and blocked with 5% bovine serum albumin at room temperature for 1 h. This was followed by overnight probing with primary antibody at 4°C and incubation with anti-mouse or anti-rabbit horseradish peroxidase-linked IgG secondary antibodies for 1 h. Blots were visualized using a charge-coupled-device imager detector (Bio-Rad, USA) after enhanced chemiluminescence detection (Amersham, UK). The primary antibodies used include anti-229E nucleocapsid mouse monoclonal (Eurofins Ingenasa, Spain) and anti-229E spike rabbit polyclonal antibodies (Genscript, USA).

### Electron microscopy.

Virus samples were PEG precipitated and subjected to centrifugation on a 20% sucrose cushion. Purified viruses were resuspended in NTE buffer and fixed with 25% glutaraldehyde. Fixed virus samples were loaded onto Formvar carbon-coated copper grids and were negative stained with 1% phosphotungstic acid for 1 min. Virions were visualized with the JOELJEM-1400 Flash transmission electron microscope operated at 100 kV.

### Statistics.

Statistical analyses were performed using the unpaired two-tailed parametric Student’s *t* test in GraphPad Prism 9.

### Data availability.

The full genome consensus sequences for C2R10 viruses are available as an alignment on Genbank (OQ920097-OQ920101).
