# Supplementary material for: Spike-Independent Infection of Human Coronavirus 229E in Bat Cells
Source: Microbiol Spectr. 2023 May 18;11(3):e03483-22. doi: 10.1128/spectrum.03483-22 (PMC10269751; doi:10.1128/spectrum.03483-22)
Supplement: Supplemental file 1 — Supplemental material. Download spectrum.03483-22-s0001.pdf, PDF file, 4.5 MB [file spectrum.03483-22-s0001.pdf]

Spike independent infection of human coronavirus 229E in bat cells

Marcus G Mah,[a*] Martin Linster,[a*]# Dolyce HW Low,[a] Zhuang Yan,[a] Jayanthi Jayakumar,[a] Firdaus Samsudin,[b] Foong Ying Wong,[a] Peter J Bond[b,c] Ian H Mendenhall,[a] Yvonne CF Su,[a] Gavin JD Smith[a,d,e,f]#

[*]The authors contributed equally.

#Corresponding authors.

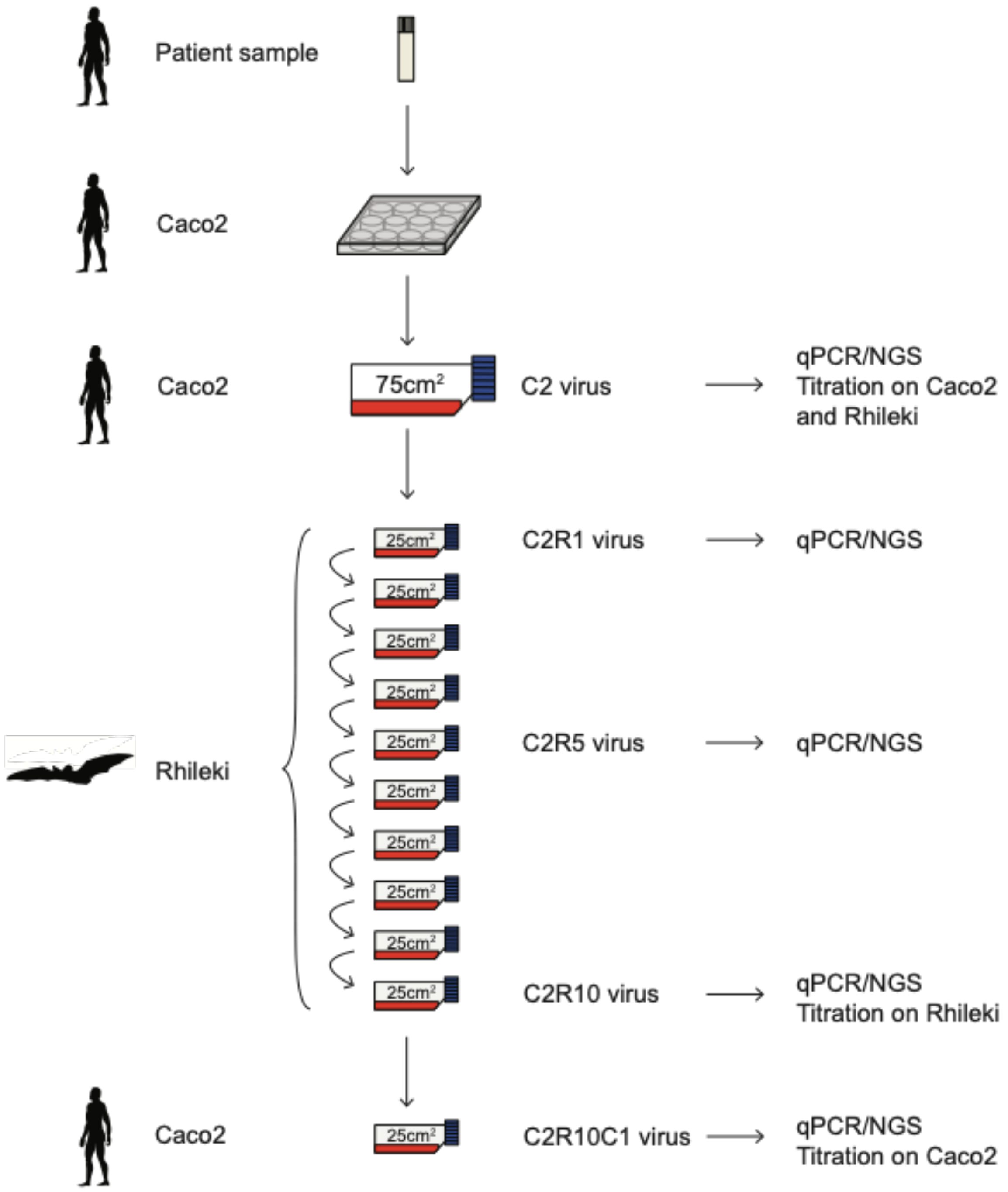

Figure S1. Schematic of virus passage experiments. Succession of the virus cultures, nomenclature of viruses, and tests performed on individual isolates.

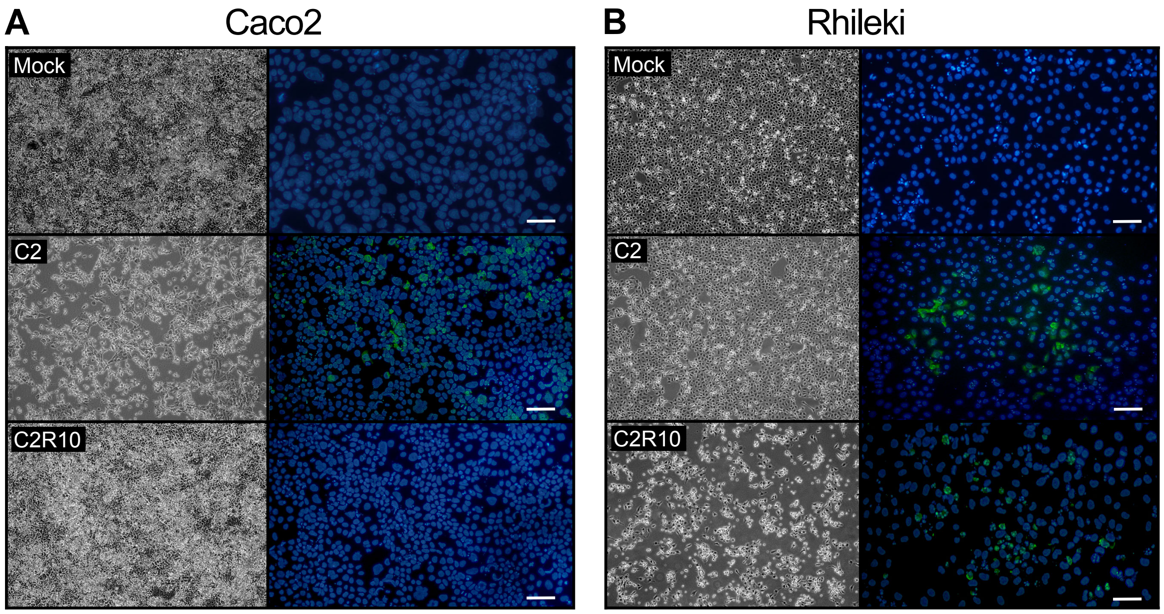

Figure S2. Microscopy and immunofluorescence of Caco2 and Rhileki cells infected with C2 or C2R10 viruses. (A) Bright light and immunofluorescence microscopy of Caco2 cells inoculated with C2, C2R10 virus or mock. (B) Bright light and immunofluorescence microscopy of Rhileki cells inoculated with C2, C2R10 virus or mock. 229E viral nucleocapsid staining (green) and nuclear staining (blue). White scale bars correspond to 100 µm.

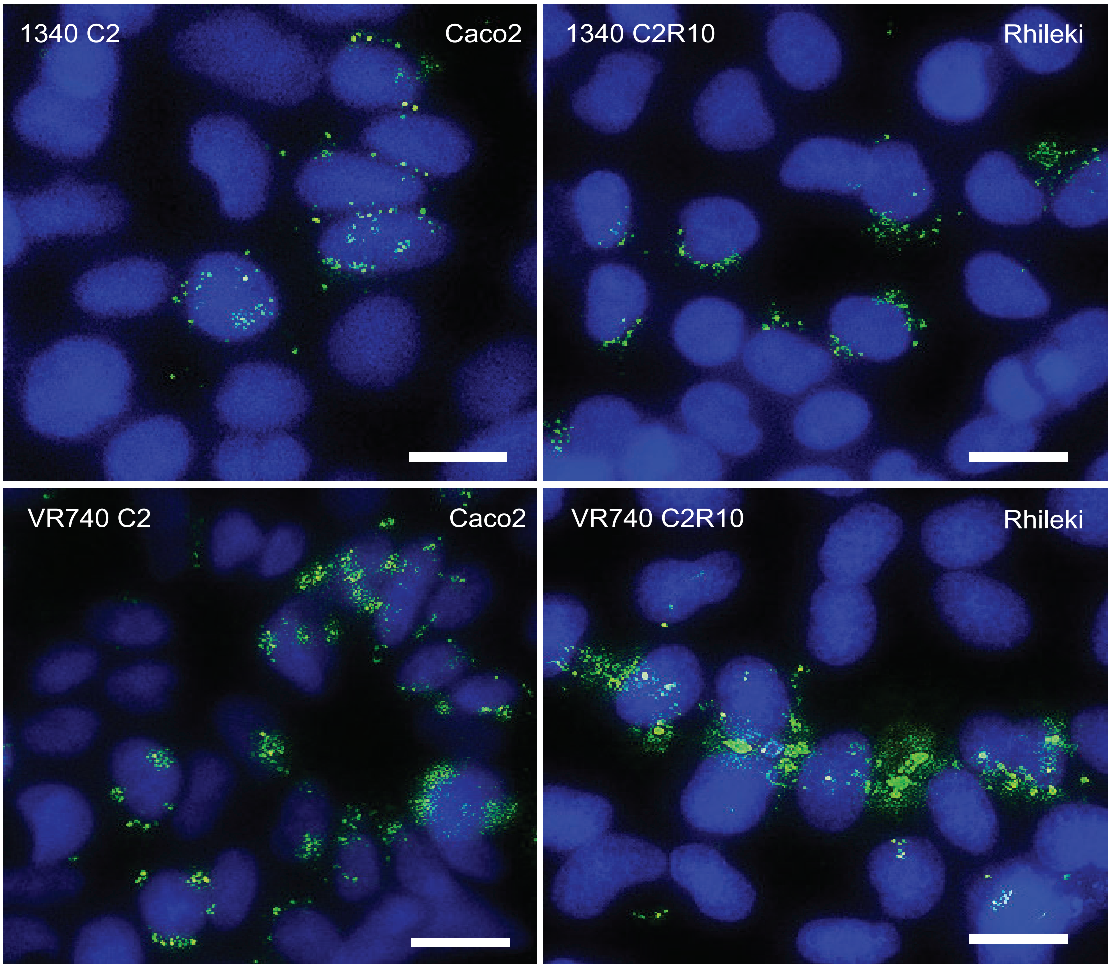

Figure S3. Double-stranded RNA staining of Caco2 and Rhileki cells. Representative images of Caco2 cells inoculated with selected C2 viruses and Rhileki cells inoculated with two C2R10 viruses. Double-stranded RNA intermediates (green) and nuclear staining (blue) visualized at 1 day post inoculation. White scale bars correspond to 20µm.

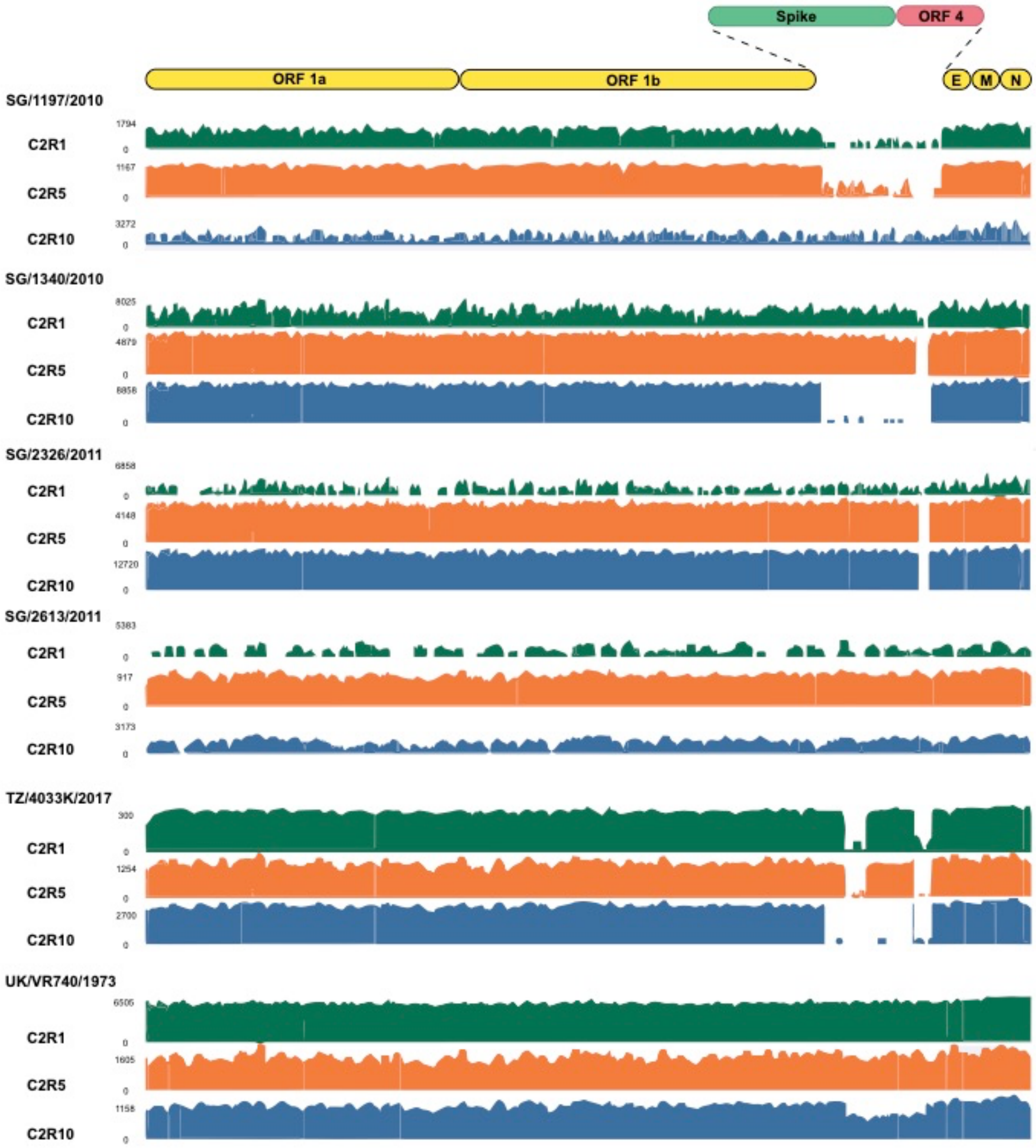

Figure S4. Next-generation sequencing. Full genome coverage plot of reads obtained from Rhileki cells infected with C2R1, C2R5, or C2R10 viruses.

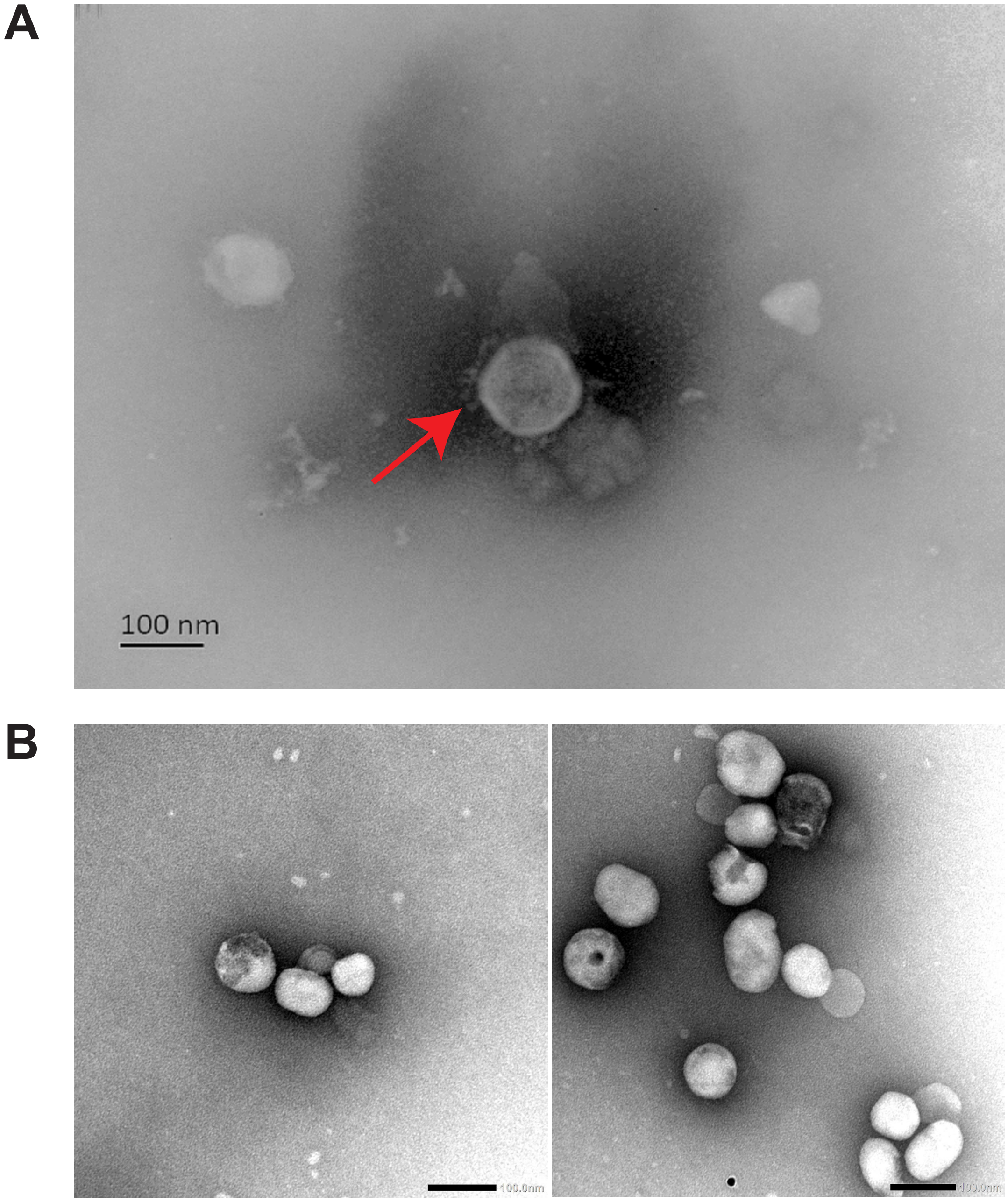

Figure S5. Electron microscopy. Negative staining and imaging of (A) C2 and (B) C2R10 virus particles. Red arrow indicates the spike protein crown-like appearance. Scale bars are present beneath each image.

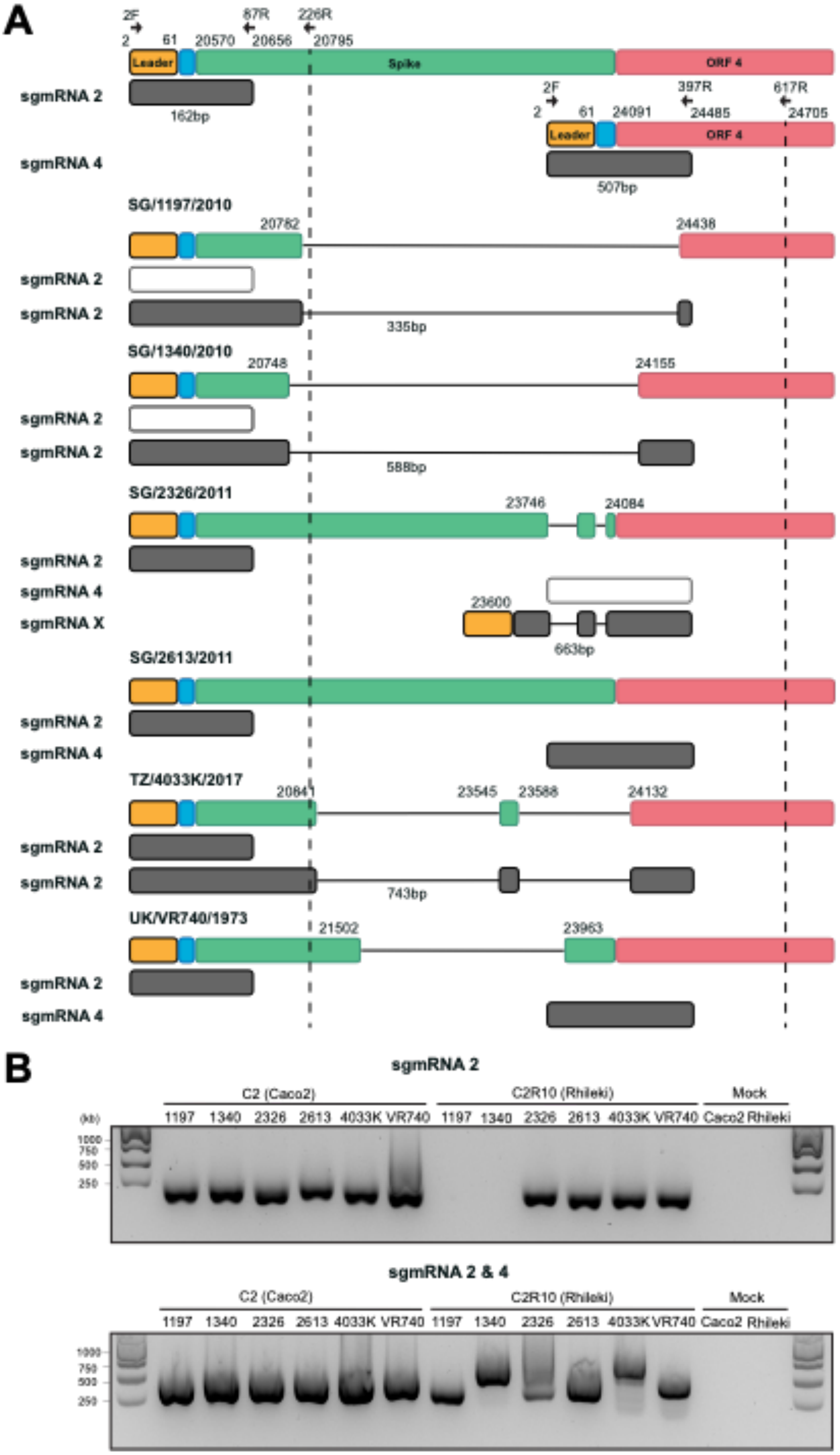

A
2F
87R
226R
2
61
20570
20656
20795
Leader
Spike
ORF 4
sgmRNA 2
162bp
397R
617R
24091
24485
24705
sgmRNA 4
507bp
SG/1197/2010
20782
24438
335bp
SG/1340/2010
20748
24155
588bp
SG/2326/2011
23746
24084
sgmRNA X
23600
663bp
SG/2613/2011
TZ/4033K/2017
20841
23545
23588
24132
743bp
UK/VR740/1973
21502
23963
B
sgmRNA 2
C2 (Caco2)
C2R10 (Rhileki)
Mock
1197 1340 2326 2613 4033K VR740 1197 1340 2326 2613 4033K VR740 Caco2 Rhileki
(kb)
1000
750
500
250
sgmRNA 2 & 4

Figure S6. Schematic describing the detection of 229E subgenomic mRNA 2 and 4 in total RNA extracted from C2R10 virus infected Rhileki cells. (A) Filled rectangles represent nucleotides at the 5’ end of sgmRNA 2 and 4 species. The leader sequence, leader-body junction (LBJ), spike, and ORF 4 reading frames are represented in orange, blue, green, and red, respectively. Primers targeting the LBJ of both sgmRNA 2 and 4 are denoted by arrows. PCR products are indicated by dark gray rectangles for sgmRNA 2 (162bp) and sgmRNA 4 (507bp), whereas white rectangles represent the absence of an expected PCR product. Rectangles of varying sizes represent the observed sequences in spike and ORF4 regions transcribed from genomic RNA. Horizontal black lines indicate deletions. (B) PCR amplification and gel electrophoresis of PCR products described in (A). Total RNA extracted Caco2 cells infected with C2 viruses were used for comparison purposes.

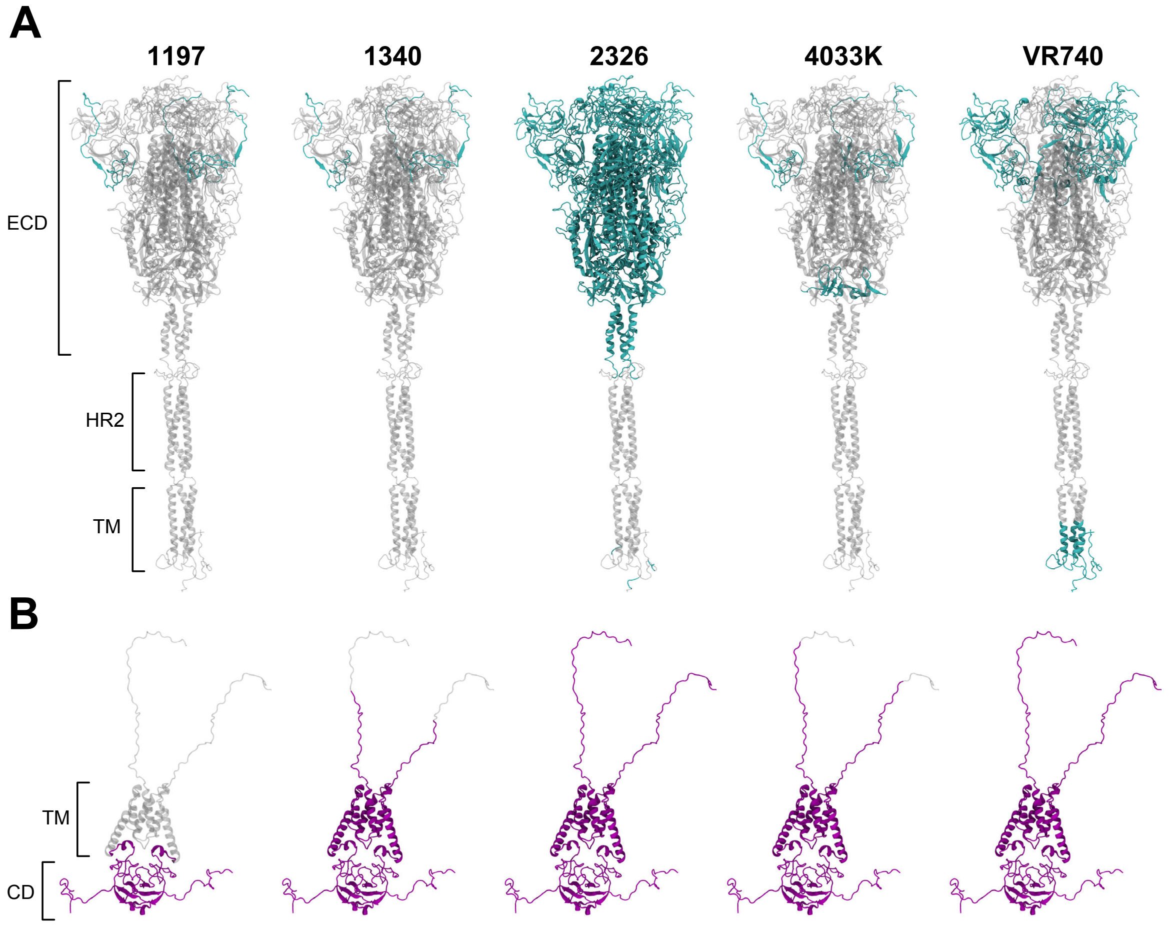

Figure S7. Modelling of deleted regions of the spike and ORF4 proteins of C2R10. (A) Full-length model of 229E spike protein built using SARS-CoV-2 spike protein as a structural template. The model is shown in cartoon representation, with the deleted regions coloured in grey, and remaining spike protein in cyan. (B) Full-length model of ORF4 protein, built using the cryo-EM structure of the SARS-CoV-2 ORF3a ion channel (PDB: 7KJR). Deleted regions are shown in grey, and the remaining protein shown in purple. Domains are labelled as ECD (ectodomain), HR2 (heptad repeat 2 domain), TM (transmembrane domain), and CD (cytoplasmic domain).

Table S1. Primers used in this study.

Name	Directionality	Sequence	Usage
1197 P5 Del F	Forward	CGGCTGTGTTGGTCATTCG	Deletion verification
1197 P5 Del R	Reverse	GGCTGAGGTCTTTATCAATACG	Deletion verification
1340 P5 Del F	Forward	CGTATTCCTACCATAGC	Deletion verification
1340 P5 Del R	Reverse	AGATCTGCCACGGTGTGATC	Deletion verification
2326 P5 Del F	Forward	GCTCTGAGTTGCAAACTATTGTGC	Deletion verification
2326 P5 Del R	Reverse	CGTGAAACTTCAGCACTAAC	Deletion verification
T140 P5 Del F	Forward	CTTGTTAGGAGTGGTAAGTTGC	Deletion verification
T140 P5 Del R	Reverse	GCAAGTTGAAGGTAACAGTGCC	Deletion verification
T140 P10 Del F	Forward	GCATATGCCTTGTTGCATATTGC	Deletion verification
T140 P10 Del R	Reverse	CGCTAGCAAGTTGAAGGTAACAGTGC	Deletion verification
T140 Del R	Reverse	GCACAACCCAGACCACG	Deletion verification
T4033 P5 Del F	Forward	CTCCATACTGTCTTGCTGC	Deletion verification
T4033 P5 Del R	Reverse	GCAGATCTGCCACGGTGTGAT	Deletion verification
T4033 P10 Del F	Forward	GGTTATATACCCTCCAACTTTGC	Deletion verification
T4033 P1-1 Del F	Forward	AGACTGAGATGTGACCAGC	Deletion verification
T4033 P1-2 Del F	Forward	TTCGTGCTTCCAGACAGCTTGC	Deletion verification
T4033 P1-1 Del R	Reverse	GCATGGCGCTATTTCTTAAGGC	Deletion verification
T4033 P10 Del-2 F	Forward	CGCATAATGTTTGAACCACG	Deletion verification
2 F	Forward	CTTAAGTACCTTATCTATCTACAGATA	sgmRNA 2/4 PCR 1/2
226 R	Reverse	GCTGAAAACTCCTCACAACACC	sgmRNA 2 cDNA
158 R	Reverse	GCAAAGTTGGAGGGTATATAACC	sgmRNA 2 PCR 1
87 R	Reverse	GCAAACAGAGTGACTAGTGTTCGTC	sgmRNA 2 PCR 2
617 R	Reverse	GCTGGGTGTTCACAAAACAAGTATA	sgmRNA 4 cDNA
520 R	Reverse	CGTACAAATCGTTAGTTGAGAG	sgmRNA 4 PCR 1
397 R	Reverse	GCACATAGCAAAGTGTGGTTAC	sgmRNA 4 PCR 2

Table S2. Assembly and statistics of NGS reads including reads mapping in deleted areas.

		SG/1197/2010	SG/1340/2010	SG/2326/2011	TZ/4033K/2017	UK/VR740/1973
Deletion present?		Y	N	N	Y	N
Size of deletion (nt)		2878	-	-	449	-
# of C2R1 NGS reads mapping to	C2 virus consensus	348	1114	102	12836	29608
	C2R1 consensus	344	1114	102	12701	29608
	Deleted region	4/348 (1.15%)	NA	NA	135/12836 (1.05%)	NA
Ratio of reads relative to size of deletion		$3.9\text{x}10^{-6}$	NA	NA	$3.9\text{x}10^{-5}$	NA
Deletion present?		Y	Y	Y	Y	N
Size of deletion (nt)		3656	443	324	1207	-
# of C2R5 NGS reads mapping to	C2 virus consensus	2897	48069	44157	22486	30839
	C2R5 consensus	2877	48862	43840	22175	30839
	Deleted region	20/2897 (0.69%)	207/49069 (0.65%)	317/44157 (0.72%)	311/22486 (1.38%)	NA
Ratio of reads relative to size of deletion		$1.9\text{x}10^{-6}$	$9.5\text{x}10^{-6}$	$2.2\text{x}10^{-5}$	$1.1\text{x}10^{-5}$	NA
Deletion present?		Y	Y	Y	Y	Y
Size of deletion (nt)		3656	3407	324	3248	2461
# of C2R10 NGS reads mapping to	C2 virus consensus	ND	64463	51642	70566	17096
	C2R10 consensus	ND	63981	51205	63250	16644
	Deleted region	ND	482/64464 (0.74%)	437/51642 (0.85%)	1316/70566 (1.86%)	452/17096 (2.64%)
Ratio of reads relative to size of deletion		ND	$2.2\text{x}10^{-6}$	$2.6\text{x}10^{-5}$	$5.7\text{x}10^{-6}$	$1.1\text{x}10^{-5}$

*ND – Not done due to low coverage

*NA – Not applicable

Table S3. Synonymous and non-synonymous mutations in virus from passages 1, 5, and 10.

Isolate	Gene	Nucleotide mutation	Amino acid mutation	Mutation	C2	C2R1	C2R5	C2R10
SG/1340/2010	NSP3	A4854G	D621G	Non-synonymous	D	100% D	65.5% G	100% G
	NSP10	C12249T	T46I	Non-synonymous	T	47% T	64.0% I	100% I
SG/2326/2011	NSP3	A4854G	D621G	Non-synonymous	D	100% D	100% D	100% G
	NSP15	T19179G	-	Synonymous	T	100% G	95.0% G	100% G
	Membrane	C25177T	L54F	Non-synonymous	L	100% L	100% F	100% F
	Membrane	T25258C	F81L	Non-synonymous	F	100% F	100% F	100% L
SG/2613/2011	NSP8	C11462T	H88Y	Non-synonymous	H	100% Y	100% Y	100% Y
	Spike	C20603T	L5F	Non-synonymous	L	100% L	100% F	100% F
	ORF4	C24200T	Q30*	Non-synonymous	Q	100% *	100% *	100% *
	ORF4	G24423T	G104V	Non-synonymous	G	100% G	96.0% G	100% V
TZ/4033K/2017	NSP2	C2954T	-	Synonymous	C	100% T	100% T	100% T
	NSP3	C7074T	S1361F	Non-synonymous	S	100% F	100% F	100% F
	Membrane	C25177T	L54F	Non-synonymous	L	100% L	100% L	100% F
	Nucleocapsid	T26118C	V137A	Non-synonymous	V	100% V	97.1% V	100% A
UK/VR740/1973	NSP2	C712T	-	Synonymous	C	100% T	72.4% T	100% T
	NSP2	C1083A	S153Y	Non-synonymous	S	100% S	100% S	86.9% Y
	NSP3	A4907G	I634V	Non-synonymous	I	62.05% I	100% V	100% V
	NSP4	T7848C	M28T	Non-synonymous	M	51.7% T	100% T	100% T

*SG/1197/2010 was excluded due to low coverage
